# Maternal adverse childhood experiences before pregnancy are associated with epigenetic aging changes in their children

**DOI:** 10.18632/aging.203776

**Published:** 2021-12-18

**Authors:** Jamaji C. Nwanaji-Enwerem, Lars Van Der Laan, Katherine Kogut, Brenda Eskenazi, Nina Holland, Julianna Deardorff, Andres Cardenas

**Affiliations:** 1 Gangarosa Department of Environmental Health, Emory Rollins School of Public Health, Atlanta, GA 30322, USA; 2 Department of Emergency Medicine, Emory University School of Medicine, Atlanta, GA 30303, USA; 3 Division of Environmental Health Sciences, School of Public Health, University of California, Berkeley, CA 94720, USA; 4 Center for Computational Biology, School of Public Health, University of California, Berkeley, CA 94720, USA; 5 Center for Environmental Research of Community Health, CERCH, School of Public Health, University of California, Berkeley, CA 94720, USA; 6 Community Health Sciences Division, School of Public Health, University of California, Berkeley, CA 94720, USA

**Keywords:** ACES, epigenetic age, DNA methylation, mitotic clocks, adversity

## Abstract

Emerging research suggests associations of physical and psychosocial stressors with epigenetic aging. Although this work has included early-life exposures, data on maternal exposures and epigenetic aging of their children remain sparse. Using longitudinally collected data from the California, Salinas Valley CHAMACOS study, we examined relationships between maternal Adverse Childhood Experiences (ACEs) reported up to 18 years of life, prior to pregnancy, with eight measures (Horvath, Hannum, SkinBloodClock, Intrinsic, Extrinsic, PhenoAge, GrimAge, and DNAm telomere length) of blood leukocyte epigenetic age acceleration (EAA) in their children at ages 7, 9, and 14 years (N = 238 participants with 483 observations). After adjusting for maternal chronological age at delivery, pregnancy smoking/alcohol use, parity, child gestational age, and estimated leukocyte proportions, higher maternal ACEs were significantly associated with at least a 0.76-year increase in child Horvath and Intrinsic EAA. Higher maternal ACEs were also associated with a 0.04 kb greater DNAm estimate of telomere length of children. Overall, our data suggests that maternal preconception ACEs are associated with biological aging in their offspring in childhood and that preconception ACEs have differential relationships with EAA measures, suggesting different physiologic utilities of EEA measures. Studies are necessary to confirm these findings and to elucidate potential pathways to explain these relationships, which may include intergenerational epigenetic inheritance and persistent physical and social exposomes.

## INTRODUCTION

Adverse Childhood Experiences (ACEs) refer to negative psychosocial experiences that individuals experience during the first 18 years of life. ACEs are typically enumerated using a 10-domain framework that includes abuse (emotional, physical, sexual), neglect (emotional, physical), and household dysfunction (domestic violence, divorce, incarcerated relative, mental illness, substance abuse) [1]. In addition to the immediate harmful effects on children’s health, there is growing evidence that ACEs also have important health consequences later in life – well into adulthood [2]. Studies have identified ACEs to be a strong risk factor for cancer, cardiovascular, metabolic, and neurodegenerative disease [1, 3], with studies conducted in the United States demonstrating that four or more ACEs are associated with a 1.4 to 37.5 greater odds of some of the leading causes of death [4]. Additionally, literature has demonstrated the potential for ACEs to also impact the health of an exposed person’s offspring. For instance, studies of women exposed to ACEs describe an increased risk of preterm birth, which in turn can increase health risks for their offspring later in life [5]. Still, the mechanism by which ACEs can result in intergenerational effects remains unclear.

Toxic stress responses including activation of the hypothalamic pituitary adrenal axis and inflammation are the main mechanisms hypothesized to explain how ACEs may cause biological changes that impact health outcomes [6]. Among these various biological changes are epigenetic alterations and telomere shortening, both of which are changes also involved in human aging [7, 8]. Studies have reported associations of exposure to ACEs with telomere length, primarily shortening [9]. The few studies focusing on the relationships of ACEs with DNA methylation have shown that the differentially methylated regions associated with ACEs have strong relationships with DNA repair processes and parental health and that these alterations persist into mid-life [10, 11], but as yet no studies have examined the intergenerational relationships.

DNA methylation-based biomarkers of biological aging – also known as epigenetic aging markers – have been found to outperform single methylation loci and directly measured telomeres in predicting healthspan and lifespan [12–14]. Hence, epigenetic aging may be a useful and robust tool for improving our understanding of the intergenerational negative effects of ACEs [15]. Specifically, studying the relationships between ACEs and epigenetic aging may provide useful insights for better understanding ACE pathophysiology and could aid in the targeting and monitoring of interventions for offspring of exposed persons and other individuals most at risk.

With the advancement of high throughput epigenomic assays, specifically to measure DNA methylation, several highly accurate epigenetic clocks have been recently developed to study biological aging [16]. Namely, the Hannum [17], Horvath [12], and the SkinBloodClock [18] epigenetic biomarkers are predominantly DNA methylation-based predictors of chronological age but also serve as biomarkers of health status. The PhenoAge clock [13] is primarily considered a biomarker of health status, while the GrimAge clock [14] is an epigenetic biomarker of mortality risk. Intrinsic (IEAA) and extrinsic (EEAA) epigenetic age are derived from the Horvath and Hannum measures [19]. The IEAA measure reflects age acceleration independent of leukocyte proportions, which are known to change with chronological age. Hence, IEAA can be viewed as a metric of the intrinsic aging of cells. Conversely, EEAA incorporates intrinsic measures as well as age-dependent changes in leukocytes by upweighting the contributions of cells known to change with age (naïve cytotoxic T cells, exhausted cytotoxic T cells, and plasmablasts). Therefore, EEAA can be viewed as a measure of immune system aging. The DNAm telomere length (DNAm TL) biomarker is correlated with directly measured leukocyte TL in part reflecting cell replication rather than TL itself [20] while the epigenetic time to cancer 1/2 (EpiTOC/EpiTOC2) [21, 22] and mitotic age (MiAge) [23] are DNA methylation-based estimators of mitotic cell divisions. As suggested throughout the literature, each biomarker provides different information on DNA methylation-based biological aging.

In the present study, we examine the potential intergenerational impact of maternal ACEs in children from the Center for the Health Assessment of Mothers and Children of Salinas (CHAMACOS) cohort. We determine the relationships between ACEs experienced by mothers during their childhoods, prior to pregnancy, and epigenetic aging of their children at chronological ages 7, 9, and 14 years, using eight epigenetic aging biomarkers. By testing multiple biomarkers in concert, we can best characterize their relationships with maternal ACEs while also building a better understanding of which biomarkers perform best in pediatric populations. We hypothesized that ACEs experienced by mothers during their own childhood would accelerate epigenetic aging in their offspring, thus reflecting increased ACE-associated disease risk.

## RESULTS

### Cohort characteristics

The child and maternal study population characteristics are presented in Table 1. The study sample is Mexican-American and was composed of data from 238 individual children who provided 483 total observations across three age timepoints. Age 7, 9, and 14 years of age timepoints represented 33% (n = 157), 42% (n = 203), and 25% (n = 123) of the total observations, respectively. Across all study timepoints, 54% - 59% of the observations were from females. On average, mothers were approximately 26 years of age at delivery of their children. Mothers had a median (range) parity of 1 (0-5). Across the age timepoints, 51% - 54% of mothers reported no ACEs, 20% - 26 % reported 1-2 ACEs, and 23% - 26% reported 3+ ACEs. Most mothers reported abstaining from drinking (> 72%) and smoking (> 95%) during their pregnancies.

**Table 1:** Maternal enrollment and child characteristics for participants across the three study timepoints (Obs=483).

**Demographic variables**	**Age 7 timepoint (N=157)** **[33% total observations]**	**Age 9 timepoint (N=203)** **[42% total observations]**	**Age 14 timepoint (N=123)** **[25% total observations]**
**Child characteristics**			
Child Age (years), mean (SD) [range]	7.10 (0.25) [6.05-8.21]	9.11 (0.18) [9.00-10.08]	14.10 (0.16) [14.00-15.05]
Gestational Age (weeks), mean (SD) [range]	38.94 (1.46) [34-41]	39.02 (1.44) [33-42]	39.13 (1.40) [36-42]
Sex, N(%)			
Female	84 (54)	110 (54)	72 (59)
Male	73 (46)	93 (46)	51 (41)
Methylation Array/Platform, N(%)			
450K	0 (0)	203 (100)	64 (52)
EPIC	157 (100)	0 (0)	59 (48)
**Maternal characteristics**			
Maternal Age at Child Delivery (years), mean (SD) [range]	26.25 (4.74) [18-41]	26.73 (5.14) [18-43]	26.54 (4.52) [18-41]
Maternal Parity, median [range]	1 [0-5]	1 [0-5]	1 [0-5]
Maternal ACE Category, N(%)			
0	85 (54)	103 (51)	63 (51)
1-2	32 (20)	47 (23)	32 (26)
3+	40 (26)	53 (26)	28 (23)
Maternal Alcohol, N(%)			
Yes	41 (26)	50 (25)	35 (28)
No	116 (74)	153 (75)	88 (72)
Maternal Smoking, N(%)			
Yes	7 (4)	7 (3)	6 (5)
No	150 (96)	196 (97)	117 (95)

Data from 238 individual children. 81 children had DNA methylation data at all three timepoints.

### Epigenetic age performance

**Figure 1:**
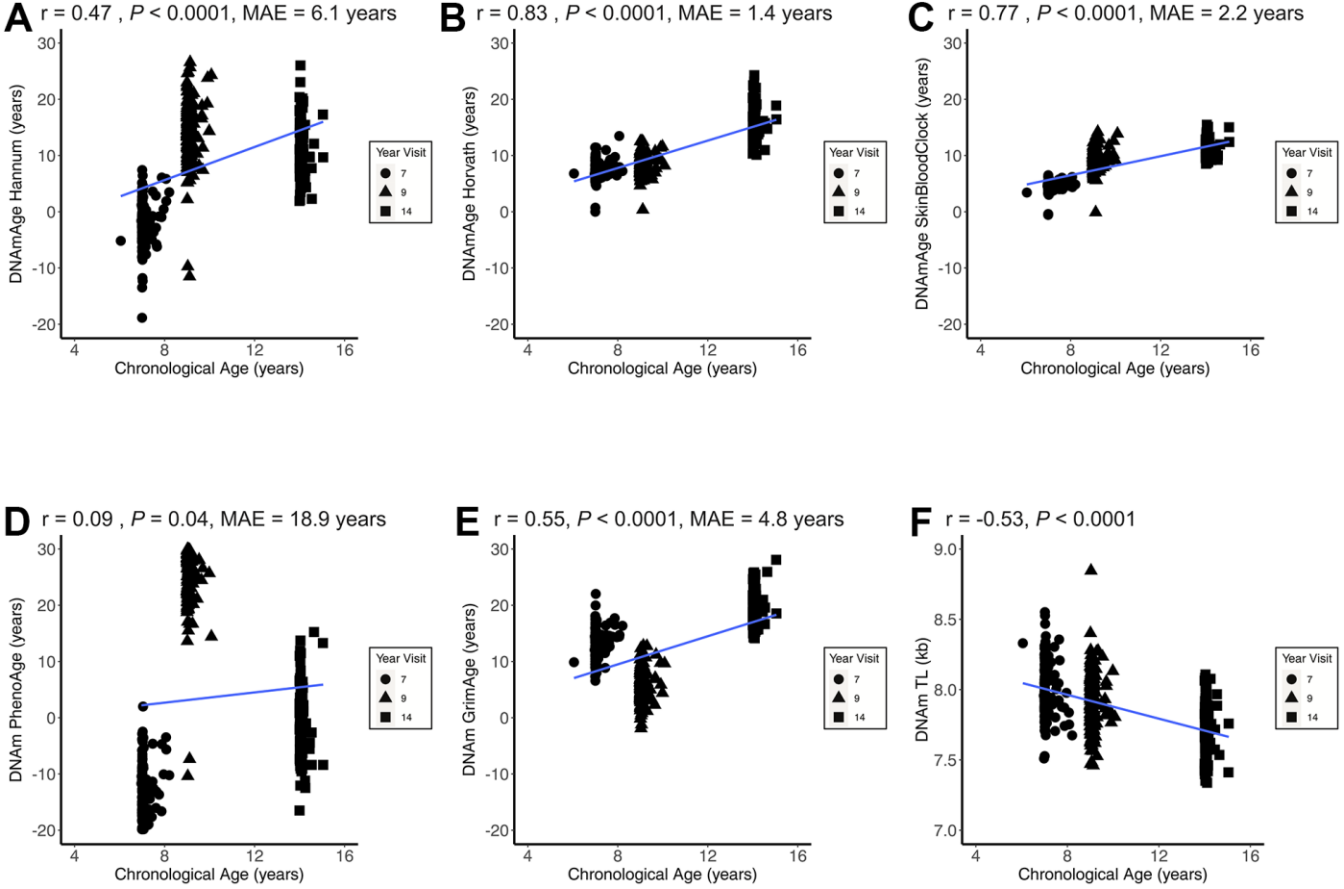
**Epigenetic age correlations with chronological age.** Figure 1 presents the child chronological age and epigenetic age correlation coefficients across all three CHAMACOS participant age timepoints (Obs = 483) for DNAmAge Hannum (**A**), DNAmAge Horvath (**B**), DNAmAge SkinBloodClock (**C**), DNAm PhenoAge (**D**), DNAm GrimAge (**E**), and DNAm TL (**F**). MAE = median absolute error.

All epigenetic clocks and the DNAmTL biomarker were significantly correlated with chronological age (Figure 1). Of the epigenetic clocks that were positively correlated with chronological age, DNAmAge Horvath (*r* = 0.83, *P* < 0.0001; MAE = 1.4 years) and the SkinBloodClock performed the best (*r* = 0.77, *P* < 0.0001; MAE=2.2 years). In the main analysis, PhenoAge performed the worst in terms of correlation and accuracy (*r* = 0.09, *P* = 0.04; MAE = 18.9 years) with very large deviations between ages. As expected, DNAm TL was the only metric negatively correlated with chronological age (*r* = -0.53, *P* < 0.0001). Compared to other epigenetic clocks, the mitotic clocks demonstrated weaker correlations (*r* < 0.3) and much greater variability relative to chronological age (Supplementary Figure 1). Supplementary Figure 2 presents the Pearson correlations coefficients between all epigenetic aging measures across all observations. The strongest positive correlation was between EAA Hannum and EEAA (*r* = 0.96, *P* < 0.0001). The strongest negative correlation was between DNAm TL and EEAA (*r* = -0.74, *P* < 0.0001).

### Associations of pre-conception maternal ACEs with child epigenetic age acceleration

Table 2 presents the associations of aggregated maternal ACEs with child EAA from fully adjusted linear mixed effects models. In these longitudinal models, with mothers reporting no ACEs as the reference, we observed significant positive associations of ACEs with EAA Horvath, IEAA, and DNAm TL. For Horvath’s EAA, mothers reporting 1-2 ACEs had on average children with 0.76-year greater EAA (95%CI: 0.24, 1.27, *P* = 0.004) when compared to children of mothers reporting no ACEs. However, children of mothers reporting 3+ ACEs did not significantly differ in Horvath’s EAA (β = 0.16, 95%CI: -0.37, 0.67, *P* = 0.56) from mother reporting no ACEs. Similarly, mothers reporting 1-2 ACEs (β = 0.80, 95%CI: 0.30, 1.30, *P* = 0.002), but not 3+ ACEs (β = 0.14, 95%CI: -0.37, 0.65, *P* = 0.59) had children with significantly greater IEAA relative to children of mothers reporting no ACEs. In contrast, children of mothers reporting 3+ ACEs had significantly longer telomere length estimates (DNAm TL) (β = 0.04, 95%CI: 0.01, 0.08, *P* = 0.009), but not children of mothers with 1-2 ACEs (β = 0.01, 95%CI: -0.02, 0.04, *P* = 0.54) when compared to children of mothers reporting no ACEs. In stratified analyses, these trends were seen in both sexes. We observed no consistent, significant associations of ACEs with EAA Hannum, SkinBloodClock, PhenoAge, GrimAge, and EEAA. Similar trends and magnitudes of associations were observed when analyses were restricted to each respective age timepoint (Supplementary Table 1). Furthermore, associations of maternal ACEs with child EAA Horvath, IEAA, and DNAm TL remained significant in main analysis models additionally adjusted for total adversity experienced by the children (Supplementary Table 2).

**Table 2:** Relationships of maternal adverse childhood experiences (ACEs) with epigenetic age acceleration (EAA) across three timepoints.

**Aging biomarker models**	**All (Obs = 483)**	***P***	**Females (Obs = 266)**	***P***	**Males (Obs = 217)**	***P***
	**Difference in DNA methylation biomarker (95% CI)**		**Difference in DNA methylation biomarker** (95% CI)*		**Difference in DNA methylation biomarker** (95% CI)*	
**EAA Hannum** *units: years*						
ACEs 0	reference	-	reference	-	reference	-
ACEs 1-2	0.61 (-0.24, 1.47)	0.16	-0.13 (-1.22, 0.96)	0.82	0.88 (-0.46, 2.22)	0.20
ACEs 3+	-0.43 (-1.31, 0.45)	0.34	-1.33 (-2.41, -0.24)	0.02	0.46 (-0.95, 1.88)	0.52
**EAA Horvath** *units: years*						
ACEs 0	reference	-	reference	-	reference	-
ACEs 1-2	0.76 (0.24, 1.27)	0.004	0.65 (-0.08, 1.38)	0.08	0.93 (0.19, 1.67)	0.01
ACEs 3+	0.16 (-0.37, 0.67)	0.56	0.13 (-0.60, 0.85)	0.73	0.38 (-0.40, 1.15)	0.34
**EAA SkinBloodClock** *units: years*						
ACEs 0	reference	-	reference	-	reference	-
ACEs 1-2	0.24 (-0.10, 0.57)	0.16	-0.03 (-0.53, 0.47)	0.90	0.42 (-0.04, 0.89)	0.08
ACEs 3+	0.09 (-0.25, 0.44)	0.59	-0.17 (-0.67, 0.33)	0.50	0.30 (-0.20, 0.79)	0.24
**Intrinsic EAA (IEAA)** *units: years*						
ACEs 0	reference	-	reference	-	reference	-
ACEs 1-2	0.80 (0.30, 1.30)	0.002	0.78 (0.09, 1.48)	0.03	0.86 (0.14, 1.58)	0.02
ACEs 3+	0.14 (-0.37, 0.65)	0.59	0.19 (-0.50, 0.89)	0.58	0.24 (-0.51, 1.00)	0.53
**Extrinsic EAA (EEAA)** *units: years*						
ACEs 0	reference	-	reference	-	reference	-
ACEs 1-2	0.49 (-0.51, 1.49)	0.33	-0.48 (-1.79, 0.82)	0.47	0.83 (-0.69, 2.35)	0.28
ACEs 3+	-0.45 (-1.47, 0.57)	0.38	-1.25 (-2.55, 0.05)	0.06	0.31 (-1.29, 1.91)	0.70
**EAA PhenoAge** *units: years*						
ACEs 0	reference	-	reference	-	reference	-
ACEs 1-2	0.42 (-0.88, 1.72)	0.52	0.72 (-1.05, 2.49)	0.42	-0.14 (-2.20, 1.91)	0.89
ACEs 3+	-0.53 (-1.86, 0.79)	0.43	-0.56 (-2.32, 1.20)	0.53	-0.58 (-2.75, 1.59)	0.60
**EAA GrimAge** *units: years*						
ACEs 0	reference	-	reference	-	reference	-
ACEs 1-2	-0.37 (-0.96, 0.23)	0.23	-0.27 (-1.14, 0.61)	0.55	-0.61 (-1.47, 0.25)	0.16
ACEs 3+	-0.55 (-1.16, 0.05)	0.07	-0.79 (-1.66, 0.07)	0.07	-0.37 (-1.27, 0.52)	0.41
**DNAm TL Age Adjusted** *units: kb*						
ACEs 0	reference	-	reference	-	reference	-
ACEs 1-2	0.01 (-0.02, 0.04)	0.54	-0.02 (-0.06, 0.03)	0.53	0.03 (-0.01, 0.07)	0.16
ACEs 3+	0.04 (0.01, 0.08)	0.009	0.04 (-0.01, 0.09)	0.08	0.04 (-0.002, 0.09)	0.06

Models adjusted for maternal chronological age at delivery, pregnancy alcohol consumption, pregnancy smoking, maternal parity, child sex, child gestational age, leukocyte abundance/proportions, and methylation platform.

*Models not adjusted for child sex.

### Associations of individual maternal ACE domains with child epigenetic age acceleration

**Figure 2:**
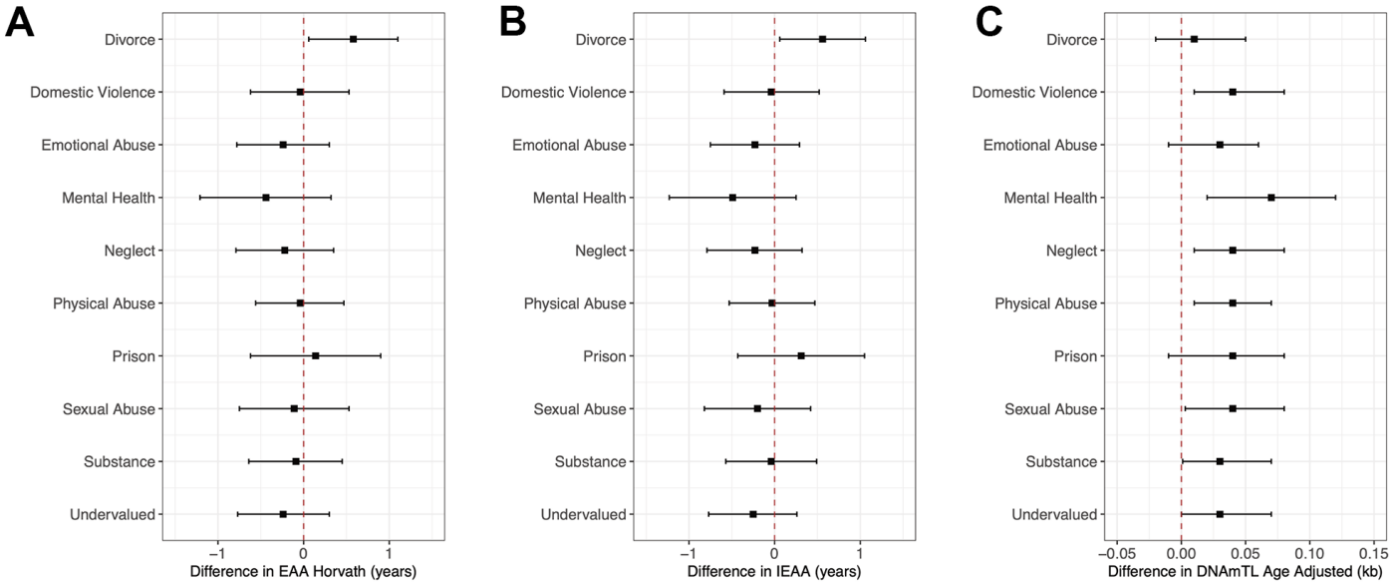
**Forest plots of model coefficients and 95% CI for methylation-based aging biomarkers by ACE domains.** Figure 2 presents forest plots of model coefficients and 95% CIs for child methylation-based age biomarkers (EAA Horvath (**A**), IEAA (**B**), and DNAmTL Age Adjusted (**C**)) across all three CHAMACOS participant age timepoints for individual ACE domains (Obs = 483). Methylation-based age biomarkers are those with statistically significant associations with cumulative ACE scores.

Figure 2 and Supplementary Table 3 present the adjusted relationships of individual ACE questions/domains with EAA biomarkers previously associated with cumulative ACEs in the present study. Of the 10 domains, having experienced a divorce or separation of parents was the only domain associated with a 0.58-year greater Horvath’s EAA (95%CI: 0.06, 1.10, *P* = 0.03). Parents’ divorce or separation was also the only domain that was individually associated with greater IEAA (β = 0.56, 95%CI: 0.06, 1.06, *P* = 0.03). Of the 10 maternal ACES domains, parents’ divorce, emotional abuse, and having a household member go to prison were the only ones that were not significantly associated with a greater DNAm TL although all estimates were positive. Effect estimates for individual domains significantly associated with DNAm TL were comparable ranging between 0.03 or 0.04 kb greater DNAm TL.

### Epigenetic mitotic clock relationship secondary analyses

The EpiTOC biomarker was not significantly correlated with chronological age (*r* = 0.05, *P* = 0.30) while weak to moderate associations were observed for the EpiTOC2 (*r* = 0.19, *P* < 0.001) and MiAge (*r* = 0.28, *P* < 0.001) biomarkers. The mitotic clocks were strongly correlated with each other (*r* > 0.65), but were weakly correlated with the other epigenetic age measures including DNAm TL (range: *r* = -0.09 to *r* = 0.16) (Supplementary Figure 1). Furthermore, there were no significant associations between ACEs and the mitotic clocks (Supplementary Table 4).

## DISCUSSION

There is growing evidence and acceptance of the premise that aging begins in youth – if not earlier [24]. Our results provide additional evidence that suggests that aging can be influenced by factors that exist before an individual’s own existence, including maternal stressors before conception. More specifically, our results show acceleration of epigenetic aging of 9 months or greater in Horvath’s clock and the IEAA biomarker in children with mothers with 1-2 ACEs but not in children with mothers with 3+ ACEs compared to those with no ACEs. However, contrary to our hypothesis of ACEs being associated with increased aging, individuals whose mothers had 3+ ACEs had significantly longer DNAm telomere estimates than those whose mothers reported no ACEs. Importantly, the individual maternal ACE domains of domestic violence, mental health, neglect, physical abuse, sexual abuse, and substance use were associated with children having a longer DNAm telomere estimate. Meanwhile, parental divorce experienced by mothers before 18 years of age appeared to be the primary driver of greater Horvath’s EAA and IEAA relationships among their children. We observed no consistent, significant associations of ACEs with other epigenetic clocks, providing evidence that different markers may be sensitive to different stressors. Although not always statistically significant (likely due to smaller sample sizes of the restricted age timepoint analyses), similar trends were observed in models of age 7, 9, and 14 years respectively.

There are a few studies examining relationships of individual stressors related to the ACEs scale with epigenetic aging, but these studies examine relationships of stressors experienced by children and epigenetic aging in children [25–27]. For instance, one study of African American youth living in Georgia, reported an association of racial discrimination experienced between ages 16 and 18 with Hannum epigenetic age acceleration at age 20. The study further demonstrated that the association between discrimination and age acceleration was ameliorated in youth with supportive family environments [25]. Work from the Avon Longitudinal Study of Parents and Children (ALSPAC) prospectively collected data on individual ACEs experienced from ages 0 to 14 years from 974 UK children in relationships to Horvath’s EAA when the children were 17 years old [28]. The authors observed that females reporting 4 or more ACEs had a 1.65-year greater Horvath’s EAA compared to girls with no ACEs. Although not statistically significant, similar trends of age acceleration were observed in females with 1-3 ACEs compared to females reporting no ACEs. No statistically significant differences were observed in males.

Our results expand on these findings by evaluating intergenerational ACE relationships of Hannum and Horvath’s EAA as well as other epigenetic aging measures including DNA methylation telomere estimates and mitotic clocks in children. Like studies of ACEs experienced directly by children, we observe epigenetic age acceleration with maternal ACE exposure. Specifically, we observed a 0.76-year to 0.80-year greater Horvath’s EAA and IEAA in children of mothers reporting 1-2 ACEs compared to those with no ACEs. Moreover, these associations were consistent and of comparable magnitude among males and females. It is notable that we observed statistically significant differences in Horvath’s EAA and IEAA among children of mothers reporting 1-2 ACEs but not in children of mothers reporting with 3+ ACEs. The number of observations with 1-2 ACEs vs 3+ ACEs were comparable, so it is unlikely that these differences are due to sample size. It is also interesting that we observed similar relationships in both male and female children. One potential explanation for the sex and 1-2 ACEs vs 3+ ACEs differences between our findings and the existing literature, is that the mechanisms that underly intergenerational ACE effects (ACEs experienced by mothers affecting children) may differ from the mechanisms that underly the adverse effects of ACEs experienced by children directly. Our main non-mutually exclusive hypotheses for how preconception maternal ACEs can impact children are two-fold. First, there may simply be intergenerational epigenetic inheritance pathways whereby stressors are encoded into the epigenome and impact the subsequent development of the fetus and child. These might involve DNA methylation mechanisms but also higher order chromatic structure. Secondly, mothers remain and rear children in the same social environments (community, home, family, and other dynamics) where they experienced ACEs earlier in their own lives. Thus, children growing up in that same environment experience the same ACEs directly. Observing statistically significant findings even when adjusting for measures of adversity in the children provides more support for the former intergenerational hypothesis. It is unclear why we observe epigenetic age relationships for 1-2 but not 3+ ACEs for Horvath’s EAA and IEAA. Potentially, there are varying thresholds for ACE relationships depending on the specific epigenetic age biomarker. It is possible that after a certain level of stress, physiological adaptation [29] reflected by markers like Horvath EAA and IEAA may occur. Nevertheless, studies to test this hypothesis and characterize such a process, if it exists, remain necessary.

It is important to highlight the association of ACEs with IEAA because this is a measure of biological aging that is informative about intrinsic cellular physiology. Unlike other epigenetic aging measures, research has demonstrated that IEAA is less sensitive to lifestyle and environmental factors and more likely to be determined by developmental processes under genetic control or metabolism that could be programmed at birth [30, 31]. This is of public health concern because it suggests that if someone is predisposed to age acceleration via IEAA, there might be little that they can do to alter this trajectory. Thus, the association of child IEAA with preconception maternal ACEs provides additional support to the hypothesis that preconception maternal ACEs may lead to intergenerational effects in part by epigenetic inheritance potentially through stress-related physiologic programming that may result in profound impacts on child health [32]. These findings can provide a molecular basis for the perpetuation of health disparities over time in marginalized populations.

Of the three epigenetic aging markers that demonstrated robust significant relationships with ACEs, we only observed a “dose-dependent” response with DNAm TL. Compared to children of mothers reporting no ACEs, those with 1-2 ACEs had 0.01 kb non-significantly longer and those with 3+ ACEs had 0.04 kb significantly longer DNAm telomere estimates. The directions of these relationships are opposite of most of the existing literature where experiencing more ACEs is associated with shorter telomeres directly measured by qPCR – canonically characterized as accelerated biological aging [9]. Although the DNAm TL biomarker is correlated with measured telomere in leukocytes (*r* = 0.41 to 0.50), and even more strongly correlated with chronological age than directly measured TL, this only indicates covariation and it is unclear if the biomarker truly reflects telomere length itself [33].

We previously reported that DNAm TL estimates do not directly mirror measured telomere length of cells [34]. There is evidence that DNAm TL better captures relationships of processes that increase cell turnover and thus may be a readout of cell proliferation as demonstrated in the initial report of this biomarker [20]. Under this hypothesis, longer DNAm TL, which is often associated with better health, may also reflect decreased cell turnover and cell cycle arrest – a well-studied active response to stressors [35, 36]. To further test this hypothesis, we conducted secondary analyses with epigenetic mitotic clocks that may also speak to cell turnover/division relationships. However, the mitotic clocks were not correlated with DNAm TL and they demonstrated no relationships with ACEs. It is important to highlight that the mitotic clocks were poorly correlated with chronological age, having large variation within this narrow age range in children (7-14 years old). Conversely, even within the small age range (<10 years) across all 3 visits, DNA methylation clocks performed extremely well. Interestingly, Horvath’s clock was the biomarker with the greatest accuracy demonstrating remarkable precision (median error of 1.4 years), which in turn was accelerated by maternal ACEs along with its derivate of intrinsic cell aging (IEAA) shown to be unaffected by environmental and lifestyle factors. Again, our results pose the possibility that preconception ACEs might program intrinsic properties of aging like metabolism.

Strengths of the present study include its prospective longitudinal design, standardized ACE characterization, and comprehensive inclusion of novel and complementary epigenetic age measures that were repeatedly collected. There are also some important limitations. First, of all epigenetic aging markers analyzed, Horvath’s measure is known to be the most accurate in children and also generalizable across most human tissues or cells [37]. This was also the case in our study and may be one reason why our results with other epigenetic aging measures are null due to lower precision and greater variability. Secondly, our primary analysis involved testing relationships with 8 epigenetic aging biomarkers and our secondary analysis evaluated relationships with 3 additional mitotic clocks. There may be some concern regarding multiple hypothesis testing, but the *P*-values for two out of three of our significant results are below the threshold (*P* < 0.005) that would be used for a strict Bonferroni correction. Third, our results are based on a well-characterized Mexican-American birth cohort with a high level of chronic adversity and may not be generalizable to all populations. Nonetheless, most existing studies of epigenetic aging have been conducted in European populations. Thus, examining these associations in other minority groups with high adversity can help build a more inclusive understanding of these relationships.

In conclusion, findings from our comprehensive analysis of ACEs and epigenetic aging in a Mexican-American cohort support the premise that maternal stressors experienced many years before conception can impact biological aging of children into adolescence, especially for epigenetic biomarkers reflective of intrinsic cell properties like metabolism and replication. Our two non-mutually exclusive hypotheses to explain our findings are: (A) that intergenerational epigenetic programming events after maternal ACE exposures and (B) that similar external environmental and social stressors have persisted throughout the mother’s and child’s life. In either instance, our findings reemphasize the importance of early life exposures on child health and the ongoing need for targeted public health interventions to address them. Additional studies will be important to further explore and define intergenerational ACE and epigenetic aging relationships.

## MATERIALS AND METHODS

### Study population

Between October 1999 and October 2000, the Center for the Health Assessment of Mothers and Children of Salinas (CHAMACOS) study recruited 601 pregnant women in the agricultural Salinas Valley of California [38]. At enrollment, women were ≤20 weeks gestation, English- or Spanish-speaking, Medicare eligible, planning to deliver at the county hospital, and attending prenatal care visits at one of six local community or hospital clinics serving this primarily Latino farmworker population. Of 601 initial enrollees of the cohort, 526 were followed to delivery of live, singleton newborns in 2000-2001. For this study we included 238 mother-child pairs with available DNA methylation data who provided samples and consent for genomic analysis. Mothers were interviewed in the 1^st^ and 2^nd^ trimesters of pregnancy and shortly after delivery and provided biological and environmental samples at these time points. Mothers and children were assessed at subsequent follow-up visits every year or two. At each visit, we have collected detailed information about their chemical, nutritional, physical, and social environments. Study activities have been conducted by well-trained, bilingual, bicultural study staff. Children from the CHAMACOS birth cohort have been shown to be particularly vulnerable to both economic and social hardships. The University of California, Berkeley Committee for the Protection of Human Subjects approved all study activities. Written, informed consent was obtained for all participating women, child verbal assent was obtained starting at age 7 years, child written assent was obtained starting at age 12 years, and child written consent was obtained at age 18 years.

### Adverse childhood experience (ACE) measurements

Mothers completed the Adverse Childhood Experiences (ACEs) survey at the study visit when their child was 18 years old. We used a slight adaptation of the ACE questionnaire first described by Felitti et al. [1], which included 10 Questions, one each on emotional abuse, physical abuse, sexual abuse, parental divorce/separation, household substance abuse, household mental health issues, household domestic violence, parental incarceration, emotional neglect, and physical neglect, yielding a total score which could range from 0 (indicating no ACEs) to 10 (indicating all ACEs). Questionnaires were translated to Spanish. Participants completed the ACEs questionnaire as part of a one-on-one interview conducted in a private room. Although all participants were encouraged to read and answer these sensitive items independently on an iPad, only 50% of women chose to do so; the remaining 50% completed answered the ACEs items aloud with an interviewer. For analyses, we categorized participants as reporting no ACEs (0), 1 to 2 ACES (1-2), and three of more individual ACEs (3+). Although many studies use 4 or more as the cutoff of high ACE exposure, a cut-off of three or more ACEs has been used in a number of previous studies to provide adequate numbers in the highest ACEs group [39–41].

### DNA methylation data processing

At child ages 7, 9, and 14 years, a phlebotomist collected child blood samples via venipuncture. Blood samples were refrigerated and transported to the University of California, Berkeley biorepository where samples without anticoagulant were separated into serum and clot and stored at -80° C until analysis. Trained study staff isolated DNA from the banked blood clot samples using QIAamp DNA Blood Maxi Kits (Qiagen, Valencia, CA) according to the manufacturer’s protocol with minor modifications, as previously described [42].

DNA aliquots of 1 μg were bisulfite converted using Zymo Bisulfite Conversion Kits (Zymo Research, Orange, CA). DNA was amplified, enzymatically fragmented, purified, and applied to the Illumina Infinium HumanMethylation450 and EPIC BeadChips (Illumina, San Diego, CA) according to the Illumina methylation protocol to measure DNA methylation. Both EPIC and 450K chips were analyzed using the Illumina Hi-Scan system. DNA methylation was measured at 485,512 CpG sites on the 450K BeadChip and 866,836 CpG sites on the EPIC BeadChip at a single nucleotide resolution for each sample. Samples were analyzed with the HumanMethylation450 array for all the samples (n=203) at age 9 and among 64 samples (52%) at age 14 years. All samples (n=157) collected at age 7 and 59 samples (48%) collected at age 14 were analyzed with the EPIC array.

Data were imported into R statistical software for preprocessing using minfi [43]. We first performed quality control at the sample level, excluding samples with overall low intensities (< 10.5) and technical duplicates. We computed detection *P* values relative to control probes and excluded probes with non-significant detection (*P *> 0.01) for 5% or more of the samples. We preprocessed our data using functional normalization [44]. We adjusted for probe-type bias using the regression on correlated probes method [45]. Finally we used ComBat from the sva [46] package to adjust for sample plate as a technical batch. We visualized the data using density distributions at all processing steps and performed PC analyses to examine the associations of methylation differences with technical, biological, and measured traits with global DNAm variation using PCA plots.

### Epigenetic age biomarkers

Epigenetic age measures were calculated from processed DNA methylation beta values after quality control. All primary analyses of epigenetic age measures were calculated using a publicly available online calculator (http://dnamage.genetics.ucla.edu). The epigenetic mitotic clocks evaluated in the secondary analysis (EpiTOC/EpiTOC2 and MiAge) were calculated using R code from https://doi.org/10.5281/zenodo.2632938 and http://www.columbia.edu/~sw2206/softwares.htm respectively. Measures of Epigenetic Age Acceleration (EAA) are defined as the residuals of regressing epigenetic age for each clock on chronological age. The EAA for each epigenetic clock then becomes independent of chronological age and positive or negative values indicate that an individual might be biologically older or younger, respectively. Intrinsic rates of mitotic cell divisions are calculated by dividing mitotic clock measurements by chronological age.

### Statistical analysis

We described mother-child pairs across the three timepoints using means, standard deviations (SDs) and ranges for continuous variables or frequencies and percentages for categorical variables. We used Pearson’s correlation to test the performance between chronological age and epigenetic aging biomarkers. For DNA methylation clocks, we computed the Median Absolute Error (MAE) in years (defined as the median absolute deviation between each epigenetic clock and chronological age) to evaluate accuracy. To test ACE associations with EAA, we analyzed EAA for each clock longitudinally using observations from all three age timepoints in childhood. We used linear mixed effects models with a random intercept for participants to account for repeated measures. Models examined associations of reported maternal ACEs (categorized as none, 1-2, or 3+) with child epigenetic age acceleration (EAA) or intrinsic rate (IR) measures of each of the eight epigenetic age biomarkers. We utilized models that included the following covariates chosen *a priori* based on existing child ACEs literature [26, 47]: maternal chronological age at delivery, pregnancy alcohol consumption, pregnancy smoking, maternal parity, child sex, child gestational age, leukocyte abundance/proportions, and methylation platform. These fully-adjusted models were repeated after stratifying our study sample by child sex.

Total adversity experienced by the children, measured as previously described [48], was available in a subset of participants and was added to models of maternal ACEs significantly associated with child epigenetic age as a sensitivity analysis. To better understand the factors driving the associations between total maternal ACEs and epigenetic aging, we built models testing associations of having each of the 10 individual ACE questions/domains (yes/no) with EAA measures significantly associated with aggregated maternal ACEs. Finally, given results of the primary analysis that suggested cell turnover (*i.e.* associations with the DNAm TL biomarker) may be responsible for some of the observed associations, we performed a secondary analysis of ACE relationships with epigenetic mitotic clocks (EpiTOC/EpiTOC2 and MiAge). All statistical analyses were performed using R Version 3.6.3 (R Core Team, Vienna, Austria) and an unadjusted *P* value < 0.05 was used as the threshold for statistical significance.

## Supplementary Material

Supplementary Figures

Supplementary Tables
