# Supplementary material for: Maternal adverse childhood experiences before pregnancy are associated with epigenetic aging changes in their children
Source: Aging (Albany NY). 2021 Dec 18;13(24):25653–69. doi: 10.18632/aging.203776 (PMC8751604; doi:10.18632/aging.203776)
Supplement: Supplementary Figures [file aging-13-203776-s001.pdf]

SUPPLEMENTARY FIGURES

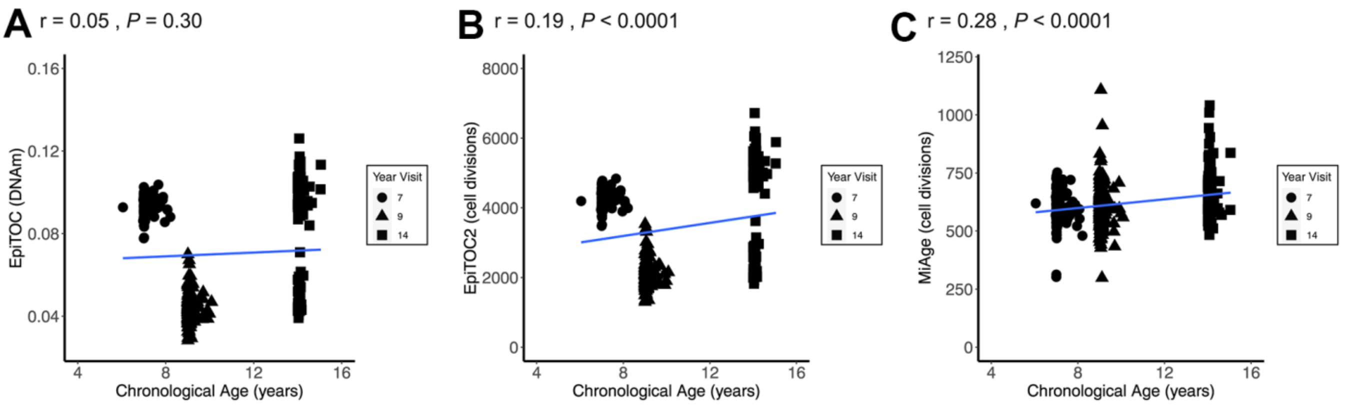

Supplementary Figure 1. Epigenetic mitotic clock correlations with chronological age. Supplementary Figure 2 presents the child chronological age and epigenetic mitotic clock correlation coefficients across all three CHAMACOS participant age timepoints (Obs = 483) for EpiTOC (**A**), EpiTOC2 (**B**), and MiAge (**C**).

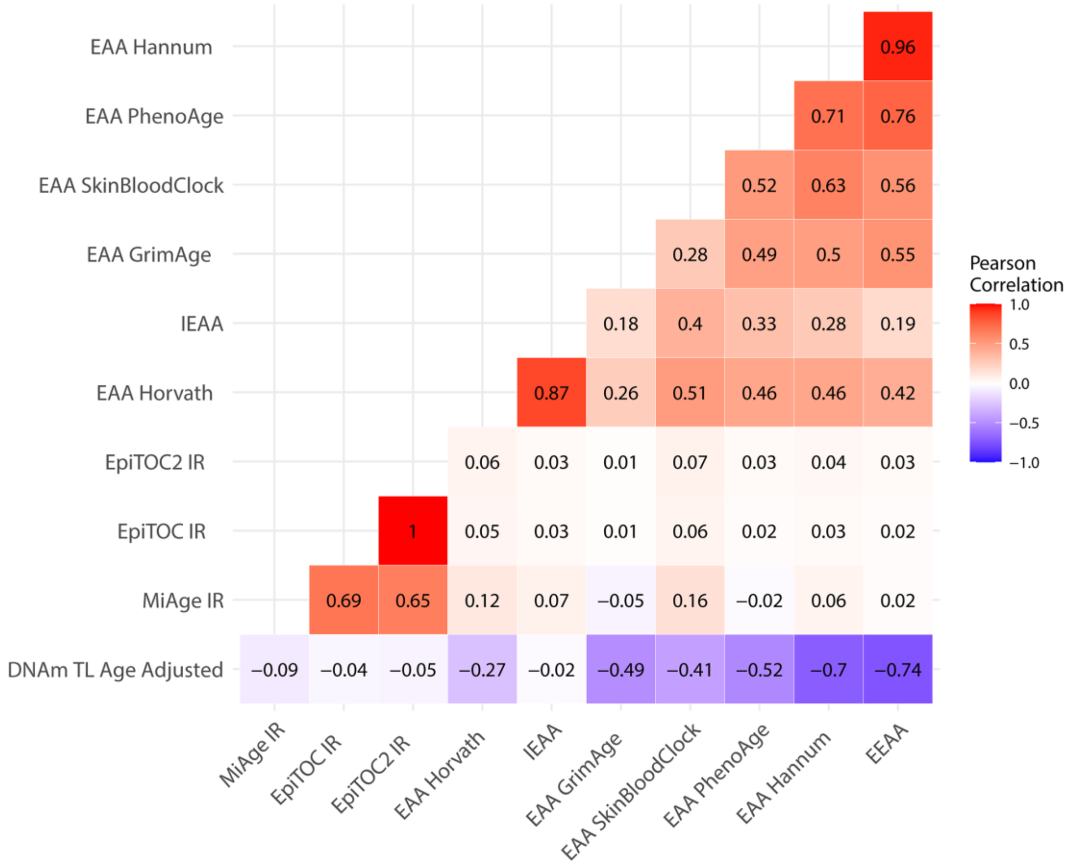

Supplementary Figure 2. Heatmap of Pearson correlation coefficients for methylation-based aging biomarkers. Supplementary Figure 2 presents Pearson correlation coefficients for child methylation-based age biomarkers across all three CHAMACOS participant age timepoints (Obs = 483).
