# Supplementary material for: Maternal adverse childhood experiences before pregnancy are associated with epigenetic aging changes in their children
Source: Aging (Albany NY). 2021 Dec 18;13(24):25653–69. doi: 10.18632/aging.203776 (PMC8751604; doi:10.18632/aging.203776)
Supplement: Supplementary Tables [file aging-13-203776-s002.pdf]

SUPPLEMENTARY TABLES

Supplementary Table 1. Relationships of maternal adverse childhood experiences (ACEs) with epigenetic age acceleration (EAA) at each age timepoint.

Aging biomarker models	Age 7 timepoint (N=157) [33% total observations] Difference in DNA methylation biomarker (95% CI)	*P*	Age 9 timepoint (N=203) [42% total observations] Difference in DNA methylation biomarker (95% CI)	*P*	Age 14 timepoint (N=123) [25% total observations] Difference in DNA methylation biomarker (95% CI)*	*P*
**EAA Horvath** *units: years*						
ACEs 0	reference	-	reference	-	reference	-
ACEs 1-2	0.39 (-0.29, 1.06)	0.25	0.80 (0.28, 1.31)	0.003	0.98 (-0.19, 2.15)	0.10
ACEs 3+	0.30 (-0.34, 0.93)	0.36	-0.03 (-0.56, 0.51)	0.92	-0.32 (-1.60, 0.96)	0.62
**Intrinsic EAA (IEAA)** *units: years*						
ACEs 0	reference	-	reference	-	reference	-
ACEs 1-2	0.39 (-0.28, 1.06)	0.25	0.80 (0.29, 1.31)	0.003	0.92 (-0.23, 2.07)	0.11
ACEs 3+	0.31 (-0.32, 0.95)	0.33	-0.02 (-0.55, 0.51)	0.94	-0.37 (-1.63, 0.88)	0.56
**DNAm TL Age Adjusted** *units: kb*						
ACEs 0	reference	-	reference	-	reference	-
ACEs 1-2	0.02 (-0.03, 0.06)	0.51	0.005 (-0.03, 0.04)	0.80	-0.01 (-0.06, 0.04)	0.69
ACEs 3+	0.05 (-0.0004, 0.09)	0.05	0.05 (0.01, 0.09)	0.01	0.02 (-0.03, 0.08)	0.41

Models adjusted for maternal chronological age at delivery, pregnancy alcohol consumption, pregnancy smoking, maternal parity, child sex, child gestational age, and leukocyte abundance/proportions.
*Model additionally adjusted for methylation platform.

Supplementary Table 2. Relationships of maternal adverse childhood experiences (ACEs) with epigenetic age acceleration (EAA) across three timepoints adjusted by total child adversity (Obs = 344).

Aging biomarker models	Difference in DNA methylation biomarker (95% CI)	*P*
**EAA Horvath** *units: years*		
ACEs 0	reference	-
ACEs 1-2	0.86 (0.29, 1.43)	0.003
ACEs 3+	0.34 (-0.30, 0.98)	0.29
**Intrinsic EAA (IEAA)** *units: years*		
ACEs 0	reference	-
ACEs 1-2	0.93 (0.36, 1.49)	0.001
ACEs 3+	0.32 (-0.31, 0.95)	0.31
**DNAm TL Age Adjusted** *units: kb*		
ACEs 0	reference	-
ACEs 1-2	0.01 (-0.02, 0.05)	0.46
ACEs 3+	0.07 (0.03, 0.12)	0.001

Models adjusted for maternal chronological age at delivery, pregnancy alcohol consumption, pregnancy smoking, maternal parity, child sex, child gestational age, leukocyte abundance/proportions, and methylation platform.

Supplementary Table 3. Relationships of individual maternal adverse childhood experience (ACE) domains with epigenetic age acceleration (EAA) across three timepoints (Obs = 483).

Aging biomarker models	Difference in DNA methylation biomarker (95% CI)	*P*
**EAA Horvath**		
units: years		
Divorce	0.58 (0.06, 1.10)	0.03
Domestic Violence	-0.04 (-0.62, 0.53)	0.89
Emotional Abuse	-0.24 (-0.78, 0.30)	0.37
Mental Health	-0.44 (-1.21, 0.32)	0.26
Neglect	-0.22 (-0.79, 0.35)	0.44
Physical Abuse	-0.04 (-0.56, 0.47)	0.86
Prison	0.14 (-0.62, 0.90)	0.71
Sexual Abuse	-0.11 (-0.75, 0.53)	0.73
Substance	-0.09 (-0.64, 0.45)	0.74
Undervalued	-0.24 (-0.77, 0.30)	0.38
**Intrinsic EAA (IEAA)**		
units: years		
Divorce	0.56 (0.06, 1.06)	0.03
Domestic Violence	-0.04 (-0.59, 0.52)	0.89
Emotional Abuse	-0.23 (-0.75, 0.29)	0.39
Mental Health	-0.49 (-1.23, 0.25)	0.19
Neglect	-0.23 (-0.79, 0.32)	0.41
Physical Abuse	-0.03 (-0.53, 0.47)	0.90
Prison	0.31 (-0.43, 1.05)	0.41
Sexual Abuse	-0.20 (-0.82, 0.42)	0.52
Substance	-0.04 (-0.57, 0.49)	0.87
Undervalued	-0.25 (-0.77, 0.26)	0.33
**DNAm TL Age Adjusted**		
units: kb		
Divorce	0.01 (-0.02, 0.05)	0.43
Domestic Violence	0.04 (0.01, 0.08)	0.02
Emotional Abuse	0.03 (-0.01, 0.06)	0.12
Mental Health	0.07 (0.02, 0.12)	0.003
Neglect	0.04 (0.01, 0.08)	0.02
Physical Abuse	0.04 (0.01, 0.07)	0.01
Prison	0.04 (-0.01, 0.08)	0.13
Sexual Abuse	0.04 (0.003, 0.08)	0.04
Substance	0.03 (0.001, 0.07)	0.04
Undervalued	0.03 (-3e-5, 0.07)	0.05

Models adjusted for maternal chronological age at delivery, pregnancy alcohol consumption, pregnancy smoking, maternal parity, child sex, child gestational age, leukocyte abundance/proportions, and methylation platform.

Supplementary Table 4. Relationships of maternal adverse childhood experiences (ACEs) with epigenetic mitotic clocks across three timepoints.

Aging biomarker models	All (Obs = 483)		Females (Obs = 266)		Males (Obs = 217)	
	Difference in DNA methylation mitotic clock (95% CI)	*P*	Difference in DNA methylation mitotic clock (95% CI)*	*P*	Difference in DNA methylation mitotic clock (95% CI)*	*P*
**EpiTOC IR** *units: DNAm*						
ACEs 0	reference	-	reference	-	reference	-
ACEs 1-2	-5.0e-5 (-5.2e-4, 4.22e-4)	0.83	-1.4e-4 (8.2e-4, 5.4e-4)	0.69	1.4e-4 (-5.4e-4, 8.2e-4)	0.69
ACEs 3+	-2.0e-4 (-6.8e-4, 2.8e-4)	0.42	8.5e-5 (-5.9e-4, 7.6e-4)	0.81	-6.1e-4 (-1.3e-3, 1.1e-4)	0.10
**EpiTOC2 IR** *units: cell divisions*	*units: years*		*units: years*			
ACEs 0	reference	-	reference	-	reference	-
ACEs 1-2	-0.15 (-20.44, 20.15)	0.99	-3.09 (-32.28, 26.11)	0.84	7.45 (-21.62, 36.51)	0.62
ACEs 3+	-9.19 (-29.95, 11.56)	0.38	3.09 (-25.92, 32.10)	0.83	-25.81 (-56.79, 5.16)	0.11
**MiAge IR** *units: cell divisions*						
ACEs 0	reference	-	reference	-	reference	-
ACEs 1-2	0.58 (-2.34, 3.51)	0.69	-0.47 (-4.85, 3.90)	0.83	2.06 (-2.21, 6.34)	0.34
ACEs 3+	-1.31 (-4.30, 1.68)	0.39	-0.59 (-4.95, 3.77)	0.79	-2.74 (-7.30, 1.81)	0.24

Models adjusted for maternal chronological age at delivery, pregnancy alcohol consumption, pregnancy smoking, maternal parity, child sex, child gestational age, leukocyte abundance/proportions, and methylation platform.
*Models not adjusted for child sex.
